# Landscape Genetics Reveals Focal Transmission of a Human Macroparasite

**DOI:** 10.1371/journal.pntd.0000665

**Published:** 2010-04-20

**Authors:** Charles D. Criscione, Joel D. Anderson, Dan Sudimack, Janardan Subedi, Ram P. Upadhayay, Bharat Jha, Kimberly D. Williams, Sarah Williams-Blangero, Timothy J. C. Anderson

**Affiliations:** 1 Department of Biology, Texas A&M University, College Station, Texas, United States of America; 2 Perry R. Bass Marine Fisheries Research Station, Coastal Fisheries Division, Texas Parks and Wildlife Department, Palacios, Texas, United States of America; 3 Department of Genetics, Southwest Foundation for Biomedical Research, San Antonio, Texas, United States of America; 4 Department of Sociology and Gerontology, Miami University, Oxford, Ohio, United States of America; 5 Tribhuvan University Institute of Medicine, Majarajgung, Kathmandu, Nepal; 6 Department of Anthropology and Department of Pediatrics, Temple University, Philadelphia, Pennsylvania, United States of America; Giovanna Raso, Centre Suisse de Recherches Scientifiques, Africa

## Abstract

Macroparasite infections (e.g., helminths) remain a major human health concern. However, assessing transmission dynamics is problematic because the direct observation of macroparasite dispersal among hosts is not possible. We used a novel landscape genetics approach to examine transmission of the human roundworm *Ascaris lumbricoides* in a small human population in Jiri, Nepal. Unexpectedly, we found significant genetic structuring of parasites, indicating the presence of multiple transmission foci within a small sampling area (∼14 km^2^). We analyzed several epidemiological variables, and found that transmission is spatially autocorrelated around households and that transmission foci are stable over time despite extensive human movement. These results would not have been obtainable via a traditional epidemiological study based on worm counts alone. Our data refute the assumption that a single host population corresponds to a single parasite transmission unit, an assumption implicit in many classic models of macroparasite transmission. Newer models have shown that the metapopulation-like pattern observed in our data can adversely affect targeted control strategies aimed at community-wide impacts. Furthermore, the observed metapopulation structure and local mating patterns generate an excess of homozygotes that can accelerate the spread of recessive traits such as drug resistance. Our study illustrates how molecular analyses complement traditional epidemiological information in providing a better understanding of parasite transmission. Similar landscape genetic approaches in other macroparasite systems will be warranted if an accurate depiction of the transmission process is to be used to inform effective control strategies.

## Author Summary

Currently, knowledge of transmission patterns of human helminth parasites is based on traditional epidemiological data such as the number of parasites within hosts. Genetic markers can greatly facilitate our understanding of the transmission process because they provide an indirect means to infer dispersal. Here, we apply novel landscape genetics methods to examine the transmission dynamics of the world's most common human macroparasite, *Ascaris lumbricoides*. Specifically, we tested for both the presence of multiple transmission foci in a single human village in Nepal and the epidemiological factors associated with such infection foci. On this very local scale, we were surprised to find multiple transmission foci that were centered on households and that reinfections were occurring from the same foci. Thus, our study illustrates the utility of population genetics analyses in epidemiology. Furthermore, our study challenges current dogma by revealing fragmentation of transmission rather than homogeneous parasite mixing within a single human community. Thus, the results have important implications for drug resistance evolution and parasite control.

## Introduction

**Figure 1:**
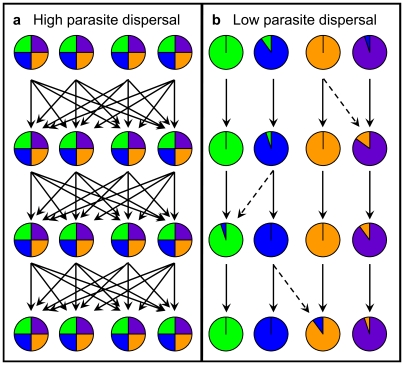
Inferring the transmission process from patterns of parasite genetic variation among hosts. Circles represent individual definitive hosts. Colors within circles are different parasite genetic variants. Dashed and solid arrows indicate limited and major paths of recruitment for parasite offspring into definitive hosts, respectively. Four generations (rows) of adult parasites are illustrated. (*A*) Parasite genetic variation is randomly distributed among hosts with a high amount of mixing among parasite offspring before recruitment into definitive hosts. This pattern indicates that hosts are randomly sampling from a common infectious pool of parasites. (*B*) Low mixing of parasite offspring (i.e., clumped transmission) predicts high genetic differentiation among individual hosts. This pattern indicates that hosts are sampling distinct infectious pools.

Effective control of infectious diseases requires knowledge of transmission dynamics. However, direct observation of parasite dispersal among hosts is almost impossible due to their biology, small size, or site of infection [1]. Molecular markers and population genetic analyses provide a useful means to examine dispersal patterns [2], [3]. For example, the rapid evolutionary rate of bacterial and viral pathogens, which have multiple generations within a host, enables the use of phylogenetic methods to infer transmission networks among hosts [4], [5]. In contrast, macroparasites typically have slower evolutionary rates and a single round of obligate sexual reproduction prior to offspring leaving the host, thus precluding the use of phylogenetic approaches to infer recent transmission events. Nevertheless, for many human helminth infections, analyses based on multilocus genotypic data and population structure can provide a powerful alternative approach to elucidate transmission patterns. Population genetic assignment methods [6] make it possible to examine if there are distinct genetic clusters of parasites (i.e., focal points of transmission) within a host population (Fig. 1) [7]. Landscape genetic analyses can then be used to test for correlations with ecological factors that may affect the distribution of genetic variation within and among these genetic clusters [8]. By incorporating the genetic clustering results with spatial, landscape, and epidemiological (e.g., host age, gender) variables, one can highlight factors that affect parasite dispersal patterns. Such molecular epidemiological data can provide a detailed understanding of parasite transmission patterns even on very local scales [2].

We employ a landscape genetics framework to examine patterns of transmission of *Ascaris lumbricoides* in a valley in the Himalayan foothills. This parasitic roundworm infects over one billion people [9], and has a direct fecal-oral lifecycle, with mating between males and females occurring in the small intestine. The study was designed to test alternative hypotheses about patterns of *Ascaris* transmission (Fig. 1). At one extreme, as long-lived eggs are present in soil and people are highly mobile, we might expect extensive mixing and a panmictic population structure. One the other hand, focal transmission hotspots could limit mixing, leading to multiple genetic clusters within a single human population. Our genetic analyses clearly demonstrate the existence of focal hotspots of transmission within this small human community. Furthermore, analyses of ecological/epidemiological factors show that these transmission hotspots are located around households. These data refute the assumption that a single host population corresponds to a single parasite transmission unit, an assumption implicit in many classic models of macroparasite transmission. Furthermore, the localized structure generates an excess of homozygous genotypes, which can accelerate the spread of recessive drug resistance alleles.

## Materials and Methods

### Ethics Statement

Written informed consent was provided by each human subject; in the case of children <18 yrs, signed or finger printed assent to participate was obtained from the individual and informed consent was given by parents or guardian. Protocols for this research were approved by the University of Texas Health Science Center Institutional Review Board in San Antonio, Texas, and by the Nepal Health Research Council, Kathmandu, Nepal.

### Sampling

Jiri, Nepal has been the subject of intensive studies on the quantitative genetics of human susceptibility to roundworm infections [10]–[12]. A description of Jiri and the sampling protocols has been reported previously [10]–[12]. Briefly, participants were given the recommended dosage (400 mg) of albendazole (Zentel; Smith Kline Beecham, London, United Kingdom). Worms were then collected over a 96-hr collection period. Roundworms were sampled from 1998 to 2003. From the 320 people (165 houses) sampled, 55 (46 houses) were sampled in two time periods. Thus, there were 375 different person-year samples and 211 house-year samples. Of these 375 person-year samples, 161 were male and 214 female; the mean age was 25.1 (range: 3–79); 188 individuals were 18 and younger and 187 were 19 and older; the mean intensity of infection was 4.48 worms per infected host (range: 1–74). Of the 165 houses sampled, the mean household size was 4.8 people (range 1–11). Molecular and genotyping methods were described previously [13], [14]. Some of the 1681 sampled worms were originally placed in formalin rather than ethanol, thus inhibiting accurate genotyping of all collected worms. For this reason, we only used worms that had complete genotypes at all 23 microsatellite loci. However, there was a strong correlation in the intensities of infection and number of worms genotyped per host (Fig. S1). Thus, our genotyping was random with respect to intensities of infection and there should be no bias with regards to parasite genetic variation.

### Identifying Genetic Structure

**Figure 2:**
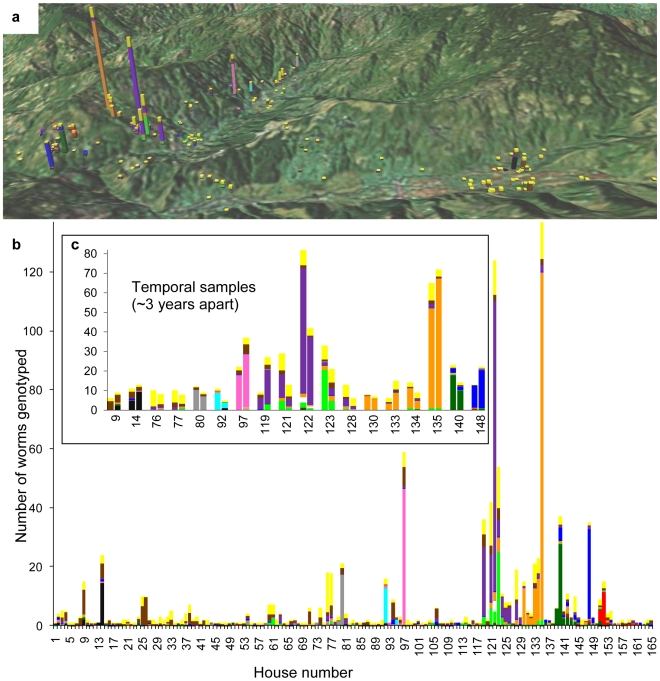
Distribution of *A. lumbricoides* genetic clusters in Jiri, Nepal. Each bar is a house and the height is the number of genotyped worms from that house. Colors within each house show the proportion of worms from the 13 core genetic clusters identified by structure. The latter was generated by summing the *Q*-values of individual worms within houses. (*A*) The geographic location of each house is illustrated over the landscape of the village. (*B*) The same information as in (*A*) but is linear to show full coloration of each house. (*C*) Displays the houses that could be tested for changes in parasite genetic composition over the two temporal samples (∼3 years apart). After correction for multiple comparisons, no household showed a significant change in parasite genetic composition. As an example to illustrate the house effect, house #97 had 59 genotyped worms (*B*), 22 and 37 from the two temporal samples (*C*). House 97 consisted of 77% of the pink genetic cluster and accounted for over 85% of the pink cluster in all the data. These results were obtained with *k* set to 15. Figure S4 shows the distribution of genetic clusters for *k* = 5.

To determine if there were foci of transmission, we used structure [15] to test for the presence of genetic clusters in the human population. In structure, we ran 10 replications of *k* populations (*k* = 1–20) with a Markov chain Monte Carlo (MCMC) burn-in of 50,000 steps and after burn-in, 100,000 steps were used to estimate parameters (Fig. S2). The no-admixture and independent allele frequency models were used. An appropriate burn-in was determined based on preliminary runs that showed stability in the estimation of the ln *P*(*D*) after 50,000 runs (e.g., see Fig. S3A). Our concern was not to determine the true value of *k*. Rather our interest was to identify evidence for genetic structure and to determine if associations between epidemiological covariates and patterns of genetic structure were consistently found at different *k*-values (Fig. 2, Fig. S4, Table 1).

**Table 1:** Results of the non-parametric multivariate analysis of variance with different distance matrices used as the dependent variable.

	structure *k* = 5	structure *k* = 15	structurama
Nested design			
Household	0.7171^***^	0.6332^***^	0.8576^***^
Host nested in household	0.0644^***^	0.0792^***^	0.0495^***^
Individual covariables			
Latitude-longitude	0.1174^***^	0.078^***^	0.1253^***^
Altitude	0.1077^***^	0.0735^***^	0.146^***^
Time	0.0073^***^	0.0052^***^	0.0089^***^
Host age	0.0055^***^	0.0038^**^	0.0099^***^
Host density	0.0528^***^	0.0345^***^	0.0938^***^
Host infection intensity	0.0774^***^	0.0455^***^	0.085^***^
Parasite sex	0.0013^ns^	0.0017^ns^	0.0018^ns^
Host sex	0.0021^ns^	0.0023^*^	0.0026^ns^
Nested design conditional on 8 covariables			
Household	0.3668^***^	0.3880^***^	0.3959^***^
Host nested in household	0.0850^***^	0.0938^***^	0.0791^***^

When the model is conditioned on the nested design, none of the covariates are significant.

ns, not significant; *, *P*<0.05; **, *P*<0.01; ***, *P* = 0.001.

### Landscape Genetics Analyses

We used non-parametric multivariate analysis of variance to determine which spatial, geographical, or epidemiological features were associated with the assignment of individual parasites to genetic clusters [16]–[19]. To achieve this, we used distlm [20] to convert the *Q*-value output of structure into a distance matrix by using a fourth root transformation with no standardization and the Bray-Curtis dissimilarity measure. Subsequently, distlm was used to perform the permutations (999) to test for significance in the non-parametric multivariate analysis of variance.

Non-parametric multivariate analysis of variance and multiple regression are well established methods to test the significance of explanatory variables on a distance matrix or a multivariate data matrix of response variables [16]–[19]. For example, in ecological studies, it is often of interest to model the environmental variables that influence the similarities among a set of samples on the basis of multivariate species abundance or percent coverage data [21]–[23]. The *Q*-value output of structure is analogous to species percent coverage data in that each cluster is like a separate species and the *Q*-value, which is similar to percent coverage data, is the genomic contribution of a population to the sample (i.e., an individual's genome). As noted above, we converted this information into a genetic distance matrix among individuals.

The novel incorporation of the individual-based genetic assignment results with a non-parametric multivariate analysis of variance enabled us to quantitatively test potential explanatory variables that could affect the underlying genetic structure. In this way, we could independently assess multiple explanatory variables without delineating populations. We applied this methodology to results from different *k* values (*k* = 5 and 15) in order to determine the robustness of the explanatory variables. The use of the *Q*-values from structure to create a distance matrix assumes that all the genetic clusters are equal in terms of genetic divergence from one another. This may not reflect biological reality. As a comparison we generated an additional distance matrix generated from structurama [24] as the genetic distance between individuals belonging to different clusters can be determined using this software. Structurama calculates these distances as the negative of the natural log of the probability that the two individuals are clustered into the same population. This probability is based on the fraction of time the two individuals were placed into the same cluster during the MCMC analysis. With structurama, our goal was to generate a genetic pairwise distance matrix among the roundworms and not to investigate the presence of genetic clusters. Hence, we ran the program at a set *k* = 5 and *k* = 12; however, both analyses produced nearly identical results so we only report results from *k* = 12. In structurama, we ran 10 Markov chains with 300,000 MCMC cycles, a sample frequency of 20, and a burn-in of 1,000.

We performed a hierarchical analysis with hierfstat [25] to test genetic structure for individual roundworms within hosts (average *F_IS_* within hosts), among hosts within households (*F_SC_*), and among households (*F_CT_*). Significance of *F_IS_* was tested in spagedi [26] with 10,000 permutations of alleles among individuals within populations. Significance of *F_SC_* and *F_CT_* was tested in hierfstat with 10,000 permutations of roundworms among hosts within households and of hosts among households, respectively. We also provide standardized measures of the *F*-statistics according to methods previously reported (Table S1) [27], [28].

**Figure 3:**
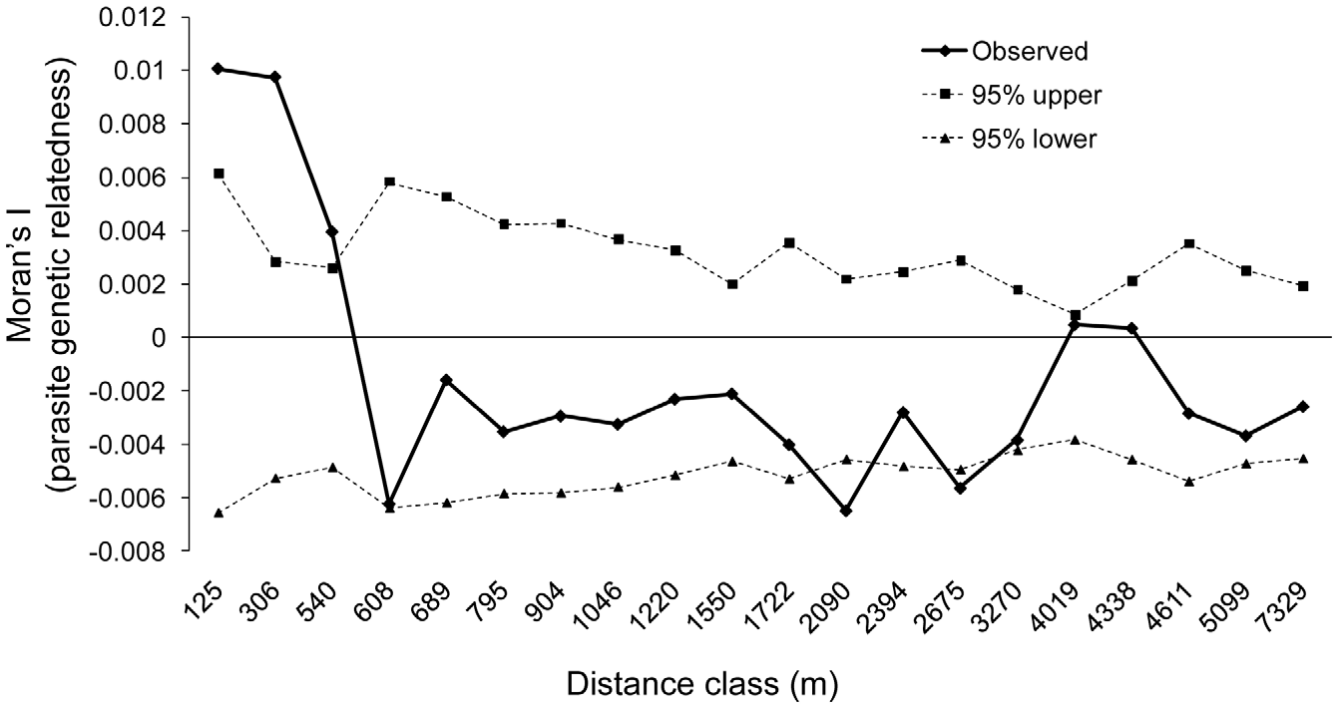
Spatial autocorrelation analysis showing nearby houses share genetically related parasites. Distance classes up to 540 m show higher parasite genetic similarity compared to values generated from random allocation of households among geographic locations (95% upper and lower confidence values). This result was also found when a single parasite was sampled from each household (Fig. S5), demonstrating that this result is not driven by a small number of heavily infected households.

A spatial autocorrelation analysis was conducted in spagedi [26] with Moran's *I* and 1,000 randomizations of geographic locations among households. Spatial intervals were selected to equalize pairwise comparisons among the distance intervals. To test the robustness of the autocorrelation with regards to the number of worms genotyped per household (or intensity of infection within household), we redid the autocorrelation analysis with a single roundworm per household. Seventy-one houses only had a single worm genotyped. For the remaining 94 we randomly included a single worm in the analysis. We created 100 new data sets by resampling roundworms with replacement. Only ten distance classes were used in order to equalize the number of comparisons among each distance class (Fig. S5). The first distance class of 473 m was approximately the same distance at which significance was detected in the full analysis (540 m; Fig. 3).

There was no effect of time in the non-parametric multivariate analysis of variance; however, we wanted to test the robustness of this result by doing direct comparisons between houses that were sampled in two time periods (∼3 years apart). For each house that we analyzed, we compared the average within-time period pairwise relatedness [29] of parasites to that expected based on the randomization of parasites among time periods. We did this test for 18 houses (Fig. 2c shows these houses based on the structure results) using spagedi. These 18 houses were analyzed because their sample sizes generated at least 900 (90%) unique permutations out of 1000 performed.

## Results

### Sampling

We collected adult *A. lumbricoides* from 320 people across 165 households that spanned an area ∼14 km^2^ in Jiri, a valley in the Himalayan foothills of Nepal (Fig. 2a). Household locations were recorded by GPS. In addition to spatial sampling, two temporal samples (∼3 years apart) were taken for a subset of the households. Of 1681 roundworms that were collected, a total of 1094 roundworms were genotyped at 23 autosomal microsatellite markers [13], [14]. Across loci, the mean expected heterozygosity was 0.71 (range: 0.19–0.95) and the mean number of alleles was 27.8 (range: 7–47). In the discussion, we provide hierarchical *F*-statistics that indicate a global deficit of heterozygosity. The distribution of the number of genotyped parasites per household (Fig. 2b) reflects the distribution of worm intensities among households as the number of genotyped worms per host significantly correlated with the actual worms burdens of individual hosts (*r* = 0.917, *P*<0.00001; Fig. S1). Thus, that only one worm was genotyped from some households reflects the natural distribution of the parasite in the human population and is not the result of poor sampling. As seen in Fig. 2b, *A. lumbricoides* in Jiri displays the typical aggregated distribution that is commonly observed among macroparasites [30], [31].

### Genetic Structure Identified on a Local Scale

We used structure [15] to test for the presence of genetic clusters in the human population (i.e., to determine if there were foci of transmission). No prior spatial or temporal information was included in the analyses. We found strong evidence for the presence of genetic structuring as indicated by an increase in maximum posterior probability (ln *P*(*D*) in the structure output) as the number of clusters (*k*) increased from *k* = 1−10, at which point an asymptote was reached and maintained to a *k* = 20 (Fig. S2). Moreover, we note that 13 core clusters, which had individual worm genetic assignments (*Q* values) that were qualitatively consistent, could be readily identified in the runs with the highest ln *P*(*D*) from *k* = 13−20. The distribution of these 13 clusters across the landscape suggested a non-random aggregation of the clusters, and thus the presence of separate transmission foci (i.e., local parasite mating units; Fig. 2). In particular, the influence of household is strikingly apparent when examining the distribution of *Q* values among the houses (i.e., distinct parasite clusters predominate individual households; Fig. 2).

We do note that the yellow genetic cluster was distributed throughout most of the households (Fig. 2). We believe this cluster is behaving as a “grab-bag” cluster (i.e., contained individuals that could not be assigned to the other clusters). A plausible explanation is that the natural aggregated parasite distribution may result in insufficient sampling of some “real” clusters, and thus there will be a low signal of genetic structure among individuals from rarer clusters. In essence, we are likely underestimating the amount of genetic structuring because the natural aggregated parasite distribution precludes sufficient sample sizes to generate genetic signatures for all “true” clusters.

### Landscape Genetics Shows Temporally Stable, Focal Transmission Centered on Households

Given the surprising visual evidence for transmission foci in such a small area, we quantitatively analyzed possible epidemiological variables that could explain the distribution of parasite genetic variation. The independent variables included a nested design (household and hosts nested within household) and eight covariables: host age, host sex, host density (number of people living in the house), elevation, geographic distance among households (latitude-longitude combined), infection intensity, parasite sex, and time. We used a nested design because individual hosts are potential pseudoreplicates of a household if hosts within households are sampling similar foci of infection. Here, we provide a novel integration of the results obtained from individual based genetic assignment methods into a non-parametric multivariate analysis of variance [17], [19]. As output, structure provides a *Q*-value list, which indicates the probability that individual roundworms are assigned to one or more clusters. We converted these data into a genetic dissimilarity matrix between pairs of worms to be used as the dependent variable [19]. To assess the robustness of the results, we created distance matrices from the structure runs with the highest ln *P*(*D*) at two disparate *k*-values (*k* = 5 and 15). We also used an additional distance matrix generated from structurama [24].

Analyses from the different distance matrices yielded similar results (Table 1). When variables were analyzed independently, household explained >63% of the variance (*P* = 0.001). Individually, each covariate always accounted for less than 15% with the geographic variables of distance and elevation always the highest (between 7–15%). When the nested design was conditioned on the eight covariates, household was still highly significant (*P* = 0.001) and explained >36%. In contrast, none of the eight covariates were significant after accounting for the nested design. Traditional measures of population genetic differentiation (e.g., *F_ST_*) are congruent with the significance of household (Table S1).

It is also noticeable that nearby houses tend to have parasites that belong to the same genetic cluster (Fig. 2). This observation is supported by a spatial autocorrelation analysis where households were the unit of interest; there was higher parasite genetic similarity than expected by chance for distance classes up to 540 m (Fig. 3). Interestingly, time had little to no impact on the genetic structure over the three year interval of sampling (Table 1). Direct comparisons between the two temporal samples for 18 houses confirmed no significant change in genetic composition over time (Fig. 2c). Only two houses, 92 and 135 (Fig. 2c), had significantly higher relatedness within time periods relative to the randomizations (*P* = 0.003 and 0.005, respectively). However, after a Bonferroni correction for 18 comparisons, neither is significant. Thus, we find little to support a temporal effect, and if present, is likely restricted to a small portion of the data set. Visually, this result is supported by comparing the distribution of *Q*-values between houses sampled in different time periods (Fig. 2c and Fig. S4c).

## Discussion

Our molecular results provide three key insights. First, there are separate transmission foci, despite the potential homogenizing influence of host movement. Second the household is the center of transmission and nearby houses share genetically related parasites, thus transmission between houses decays with distance. Third, transmission from these foci is stable over time. People are being infected and reinfected at the same source, which is associated with household units. Thus, the occurrence of parasites from two time periods in the same cluster and from the same house (Fig. 2c) indicates continuity in the transmission process. Two factors likely contribute to the observed patterns. First, the resilience of *Ascaris* eggs in the environment may lead to overlapping generations within in our sampling timeframe. Second, on average, people in Jiri tend to defecate 43.3 m (range: 2–200) from households (unpublished survey data).

Previous work on macroparasites such as *A. lumbricoides* has found that hosts with high infection intensities are often aggregated within households [32], [33]. While focal transmission around the home is commonly put forth as causative factor for this latter pattern, intensity data alone do not address the source(s) of infection. Our molecular data clearly show that transmission is focal around houses, but is unrelated to intensity of infection (i.e., houses with low infection intensities also have focal transmission). This result is supported by the spatial autocorrelation analyses when the effect of household intensities is removed (Fig. S5).

What are the implications for these separate foci of transmission? First, focal transmission may increase homozygosity due to local inbreeding or population substructure. Both processes contribute to heterozygote deficit in this system as the average inbreeding coefficient within hosts (*F_IS_*) is 0.02 (*P*<0.0001), *F_CT_* (household to the total) is 0.023 (*P*<0.0001; Table S1), and *F_IT_* (individual to the total) is 0.048 (*P*<0.0001). With an increase in homozygosity, directional selection is more efficient in driving the increase of advantageous recessive alleles [34]. Thus, focal transmission can promote the increase in frequency of possible drug resistant genes [35]. The latter is a concern as strong evidence of drug resistance in a variety of human and domestic animal macroparasites has been reported [36]. Second, while classic models of transmission incorporate heterogeneities in the transmission process, they assume a single transmission unit (implicit in the measure of a single basic reproduction number, *R_0_*) [31], [37]. Stemming from these models is the 20/80 rule (20% host population is responsible for 80% transmission), which implies targeted treatment of the heavily infected can greatly reduce transmission [31], [37]. However, recent models indicate if separate parasite populations exist in an interconnected network, then targeting high intensity infections may not improve effectiveness of control [38]. Clearly, our data do not reflect a single source for parasite infection; the transmission of *A. lumbricoides* in Jiri is better described by a metapopulation structure. Our data do not discount classic models as such dynamics may occur under different situations (e.g., a communal use of human feces for fertilizer may create a greater mixing potential of parasites and, thus a single source pool of parasites; Fig. 1a). Rather, our study highlights the need to test the assumption of a single infectious pool of parasites even on very local scales, especially given the medical and veterinary impact of many macroparasites [39], [40] (e.g., infections with *Ascaris* result in major health and economic burdens in developing countries) [9].

## Supporting Information

Figure S1 Correlation between the number of worms genotyped and the intensity of infection. The analysis was done on the 375 person-year samples (circles). The high correlation indicates that the number of genotyped worms per host-year sample is representative of the actual intensities of infection of the host-year samples.
(0.01 MB PDF)

Figure S2 Box-and-Whisker plots of the ln *P(D)* for a given *k*. The top blue horizontal lines indicate the maximum ln *P(D)* obtained out of 10 runs conducted at each *k*. Values between the inner and outer fences are plotted with asterisks. Values beyond the outer fences are plotted with empty circles. In STRUCTURE, as k increases beyond 1, an increase in the ln *P(D)* accompanied by unambiguous genetic assignment of individuals (i.e., Q-values are not split among individuals as 1/*k*) is evidence for genetic structure in the data set. We observed such patterns in our data and thus, had strong evidence for the presence of genetic clusters. As *k* increased, the variance in ln *P(D)* also increased. Many of these outlining values resulted from a fall off in the MCMC and/or the MCMC getting stuck at a suboptimal local optimum (see Fig. S3), thus indicating that these runs were not reliable. In the runs with the maximum or near maximum ln *P(D)* for *k* = 13-20, we observed consistency in the assignment of individuals to 13 core clusters. These 13 clusters were present despite the setting of *k*>13 because of the presence of empty clusters (i.e., no individuals had Q-values for these clusters) in these runs. For example, at *k* = 15 roundworms were assigned to one of 13 clusters, whereas two clusters were empty; at *k* = 20, qualitatively, the same 13 clusters were found, but there were six empty clusters and one additional cluster of only four individuals, which was split off from one of the core 13 clusters at *k* = 15.
(0.02 MB PDF)

Figure S3 Plots of the ln *P(D)* for three runs at *k* = 20. (A) A stable run that yielded the second highest ln P(D) (-92547.8) at *k* = 20. (B) A run that hits a suboptimal local optimum (ln *P(D)* = −93796.8). (C) A run showing a fall off in the MCMC (ln *P(D)* = −104212.5).
(0.07 MB PDF)

Figure S4 Distribution of *A. lumbricoides* genetic clusters in Jiri, Nepal. This figure is the same as Fig. 2 in the main text except *k* = 5.
(0.11 MB PDF)

Figure S5 Spatial autocorrelation analysis based a single roundworm per household. The above analysis represents one of the data sets that generated a significant result. Overall, 94 out of 100 of the randomly generated data sets were significant at *P*<0.05 at the first distance class of 473 m. This result indicates that the autocorrelation was robust to the number of worms genotyped per household and further supports the conclusion that transmission connectivity deceases with distance from households.
(0.03 MB PDF)

Table S1 Hierarchical *F*-statistics.
(0.03 MB DOC)
