# Supplementary figures and images for: Landscape Genetics Reveals Focal Transmission of a Human Macroparasite
Source: PLoS Negl Trop Dis. 2010 Apr 20;4(4):e665. doi: 10.1371/journal.pntd.0000665 (PMC2857643; doi:10.1371/journal.pntd.0000665)

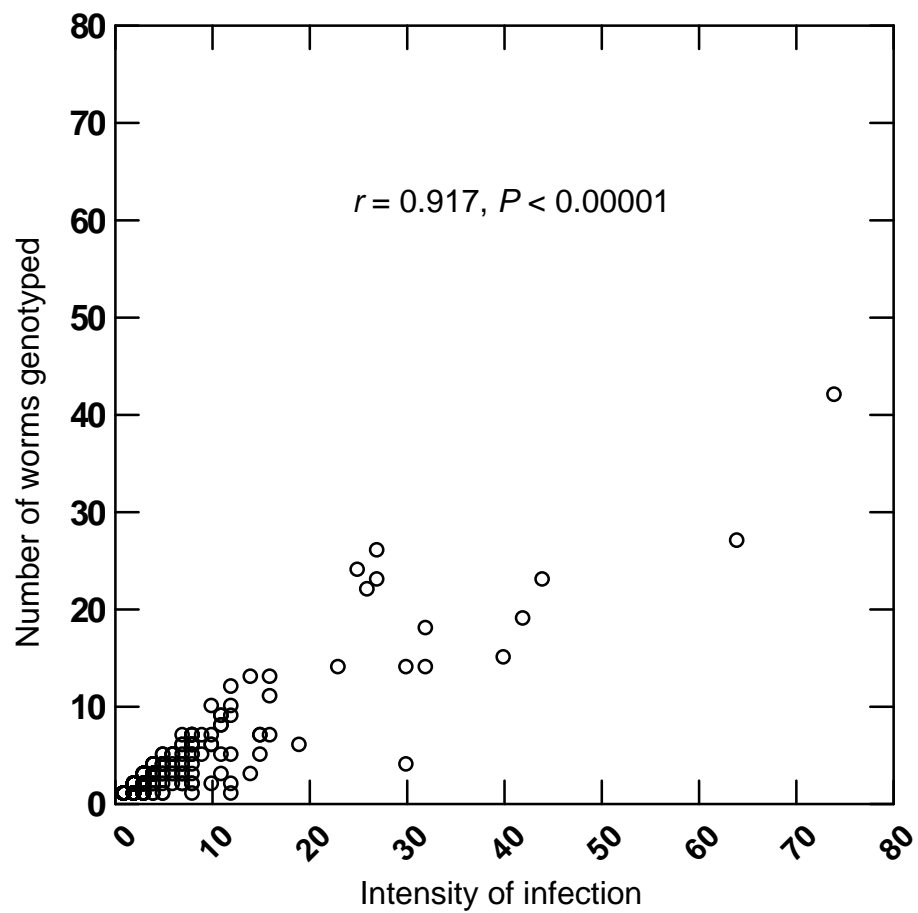

Supplement: Figure S1 — Correlation between the number of worms genotyped and the intensity of infection. The analysis was done on the 375 person-year samples (circles). The high correlation indicates that the number of genotyped worms per host-year sample is representative of the actual intensities of infection of the host-year samples. (0.01 MB PDF) [file pntd.0000665.s001.pdf]

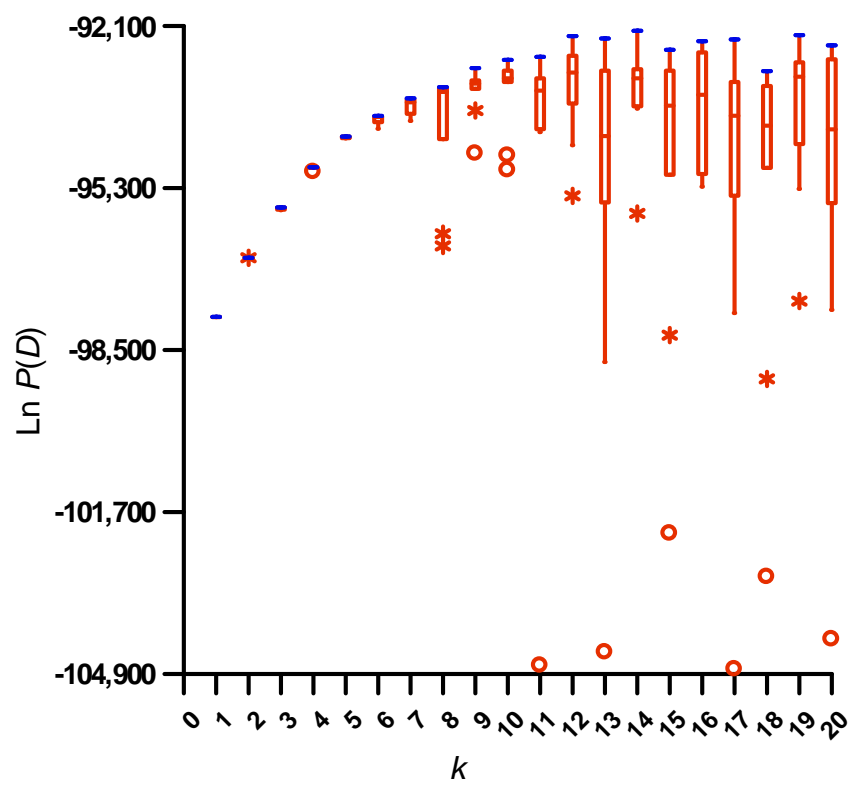

Supplement: Figure S2 — Box-and-Whisker plots of the ln P(D) for a given k. The top blue horizontal lines indicate the maximum ln P(D) obtained out of 10 runs conducted at each k. Values between the inner and outer fences are plotted with asterisks. Values beyond the outer fences are plotted with empty circles. In STRUCTURE, as k increases beyond 1, an increase in the ln P(D) accompanied by unambiguous genetic assignment of individuals (i.e., Q-values are not split among individuals as 1/k) is evidence for genetic structure in the data set. We observed such patterns in our data and thus, had strong evidence for the presence of genetic clusters. As k increased, the variance in ln P(D) also increased. Many of these outlining values resulted from a fall off in the MCMC and/or the MCMC getting stuck at a suboptimal local optimum (see Fig. S3), thus indicating that these runs were not reliable. In the runs with the maximum or near maximum ln P(D) for k = 13-20, we observed consistency in the assignment of individuals to 13 core clusters. These 13 clusters were present despite the setting of k>13 because of the presence of empty clusters (i.e., no individuals had Q-values for these clusters) in these runs. For example, at k = 15 roundworms were assigned to one of 13 clusters, whereas two clusters were empty; at k = 20, qualitatively, the same 13 clusters were found, but there were six empty clusters and one additional cluster of only four individuals, which was split off from one of the core 13 clusters at k = 15. (0.02 MB PDF) [file pntd.0000665.s002.pdf]

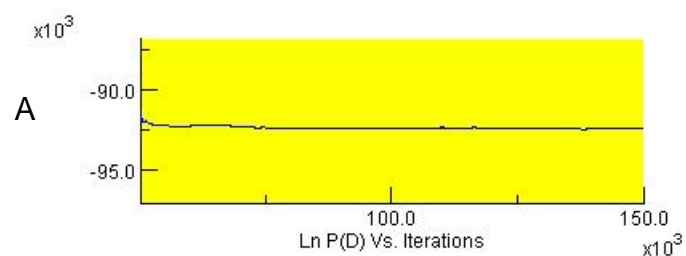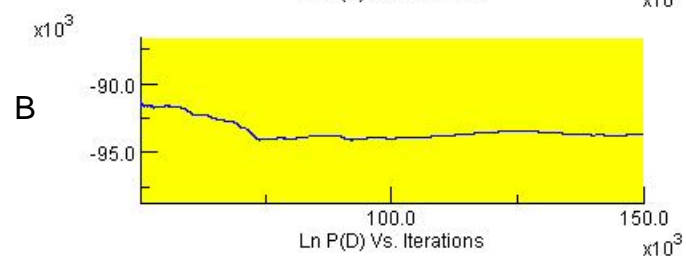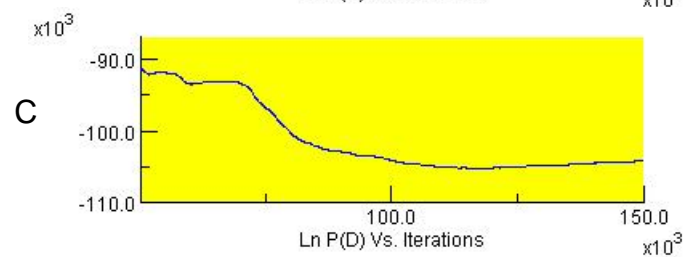

Supplement: Figure S3 — Plots of the ln P(D) for three runs at k = 20. (A) A stable run that yielded the second highest ln P(D) (-92547.8) at k = 20. (B) A run that hits a suboptimal local optimum (ln P(D) = −93796.8). (C) A run showing a fall off in the MCMC (ln P(D) = −104212.5). (0.07 MB PDF) [file pntd.0000665.s003.pdf]

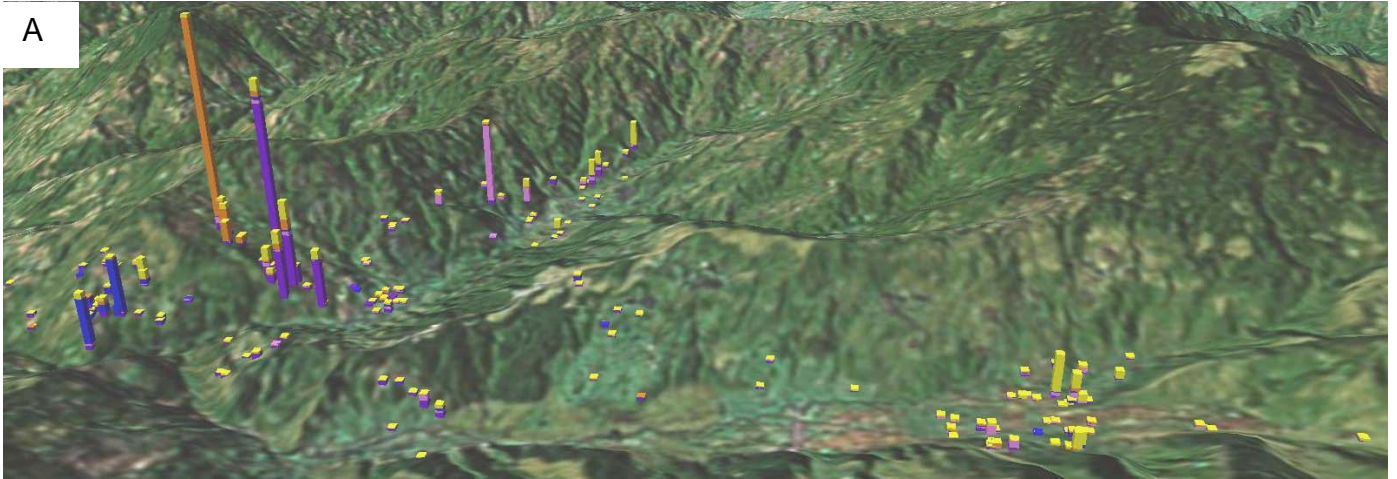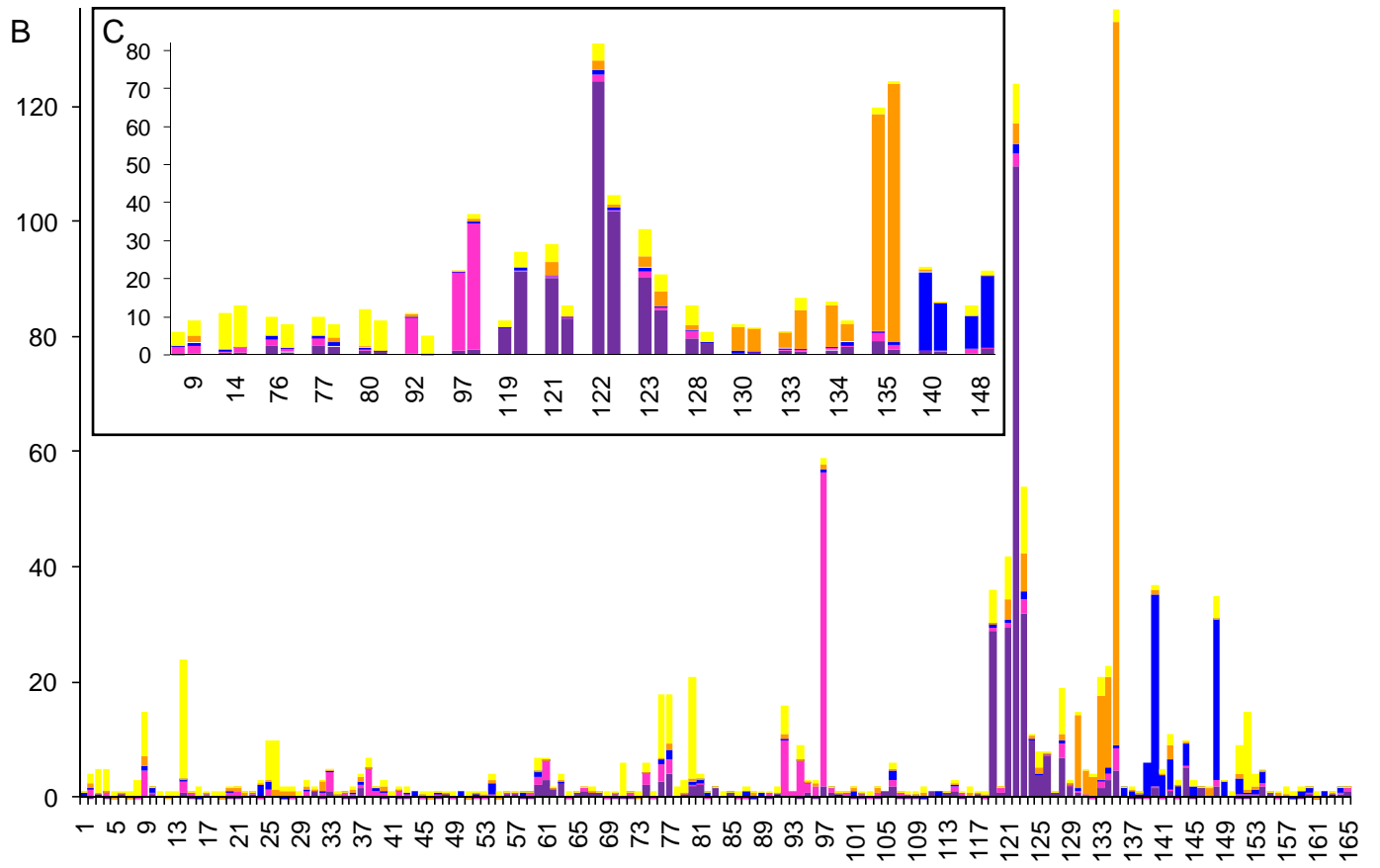

Supplement: Figure S4 — Distribution of A. lumbricoides genetic clusters in Jiri, Nepal. This figure is the same as Fig. 2 in the main text except k = 5. (0.11 MB PDF) [file pntd.0000665.s004.pdf]

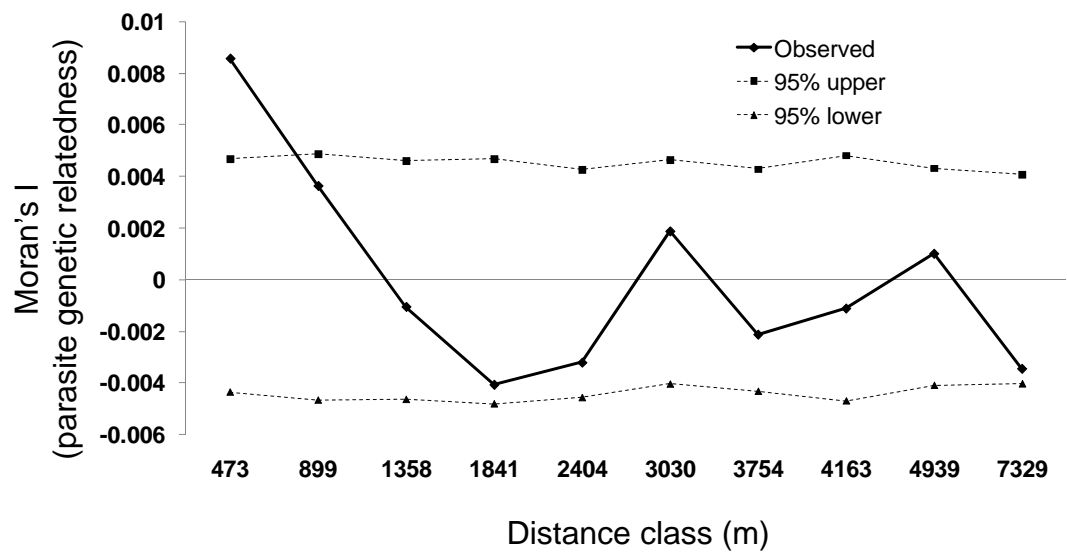

Supplement: Figure S5 — Spatial autocorrelation analysis based a single roundworm per household. The above analysis represents one of the data sets that generated a significant result. Overall, 94 out of 100 of the randomly generated data sets were significant at P<0.05 at the first distance class of 473 m. This result indicates that the autocorrelation was robust to the number of worms genotyped per household and further supports the conclusion that transmission connectivity deceases with distance from households. (0.03 MB PDF) [file pntd.0000665.s005.pdf]
