# Supplementary material for: Landscape Genetics Reveals Focal Transmission of a Human Macroparasite
Source: PLoS Negl Trop Dis. 2010 Apr 20;4(4):e665. doi: 10.1371/journal.pntd.0000665 (PMC2857643; doi:10.1371/journal.pntd.0000665)
Supplement: Table S1 — Hierarchical F-statistics. (0.03 MB DOC) [file pntd.0000665.s006.doc]

**Table S1. Hierarchical *F***-statistics.

|  | *F*-statistic | *P*-value |
| --- | --- | --- |
| *F_IS_* | 0.020 | 0.0001 |
| *F_SC_* | 0.005 | 0.0194 |
| *F_SC_’* | 0.017 | na |
| *F_CT_* | 0.023 | 0.0001 |
| *F_CT_’* | 0.077 | na |

*F_IS_* is the average inbreeding coefficient within individual hosts, *F_SC_* is individual host to household, and *F_CT_* is household to the total.

*F_SC_’* and *F_CT_’* are standardized measures of *F_SC_* and *F_CT_*, respectively.

na, significance testing is not applicable to standardized measures.
